# Rapid change of friction causes the illusion of touching a receding surface

**DOI:** 10.1098/rsif.2022.0718

**Published:** 2023-02-08

**Authors:** Jocelyn Monnoyer, Laurence Willemet, Michaël Wiertlewski

**Affiliations:** ^1^ Aix-Marseille University, CNRS, ISM, Marseille, France; ^2^ Stellantis, Human Factors Group, Velizy, France; ^3^ Cognitive Robotics Department, Delft University of Technology, Delft, The Netherlands

**Keywords:** friction, tactile perception, skin mechanics, surface haptics

## Abstract

Shortly after touching an object, humans can tactually gauge the frictional resistance of a surface. The knowledge of surface friction is paramount to tactile perception and the motor control of grasp. While potent correlations between friction and participants’ perceptual response have been found, the causal link between the friction of the surface, its evolution and its perceptual experience has yet to be demonstrated. Here, we leverage new experimental apparatus able to modify friction in real time, to show that participants can perceive sudden changes in friction when they are pressing on a surface. Surprisingly, only a reduction of the friction coefficient leads to a robust perception. High-speed imaging data indicate that the sensation is caused by a release of a latent elastic strain over a 20 ms timeframe after the activation of the friction-reduction device. This rapid change of frictional properties during initial contact is interpreted as a normal displacement of the surface, which paves the way for haptic surfaces that can produce illusions of interacting with mechanical buttons.

## 1. Introduction

Whenever our fingers physically interact with objects, tools or materials, the interaction causes our skin to deform. The deformation stimulates mechanoreceptors embedded in the tissues that signal to the brain material properties such as compliance, texture and how far the object is from sliding away. Particularly, our sense of touch can discriminate between the slipperiness of a wet soap or the grip provided by a rough surface at the first instant of touch [1,2]. The tactile sensations reinforce the internal model of our surroundings and are fundamental to properly tune our motor commands [3]. Transient events such as the crackle of a piece of bread or the subtle click of a button help us understand the dynamic behaviour of the environment [4].

However, the update of this mental model does not always reflect perfectly the physical interaction between the skin and the environment. For every interaction, the brain has to infer what is the nature of the surface being touched from an incomplete picture.

Wang and Hayward showed that unusual stimulations of the skin could fool the brain in perceiving an indentation when only local shear was applied [5,6]. Similarly, our perception of tactile speed is influenced by the texture of a surface [7]. Other illusions involve tactile and proprioception, where the compliance of an object can bias the sensation of moving the fingertip [8]. In this study, we explain the illusion that occurs when we rapidly remove the friction of the fingertip pushing on a rigid surface. Removing friction creates the illusion that the surface has moved downward (i.e. similar to the click of a keyboard). We postulate that during a simple press, the brain must decipher whether the afferent flux originates from the deformation induced by self-motion or induced from the object deformation. The distinction between the deformation of the skin coming from intrinsic or extrinsic causes is also imperfect and therefore creates the illusion of pressing a button.

We previously demonstrated that during the initial compression of the skin on a rigid surface, the friction constrains the lateral expansion of the skin [2]. In parallel, we also showed that rapid friction changes during compression lead to a clear feeling of pressing a button [9,10]. Could an intrinsic relaxation of the skin cause this sensation of key click?

When an operator is pressing on a button, elastic strain is stored in the fingertip, then suddenly released by the mechanical detent [11–13]. The interaction with buttons creates similar patterns of deformation to what happens during the rapid change of friction. Therefore, for the nervous system, the most likely explanation for this lateral relaxation of the skin would be the interaction with a button that accelerates downward.

This illusion is particularly useful for surface-haptic devices that use ultrasonic friction modulation to provide artificial tactile sensations. Ultrasonic friction modulation relies on transverse waves of the order of micrometres and outside the perceptual window of touch. However, the vibrations create a near-field acoustic levitation that reduces friction. When friction is modulated with the users’ movement, these devices can produce the illusion of shape and texture on a two-dimensional plate [14–17]. The sensation of touching a button can be triggered, not by physically displacing a button but by dynamically changing the static friction of the skin on a surface [9,10,18]. Remarkably, this behaviour arises even in the absence of sliding or lateral forces, on a static finger.

**Figure 1.**
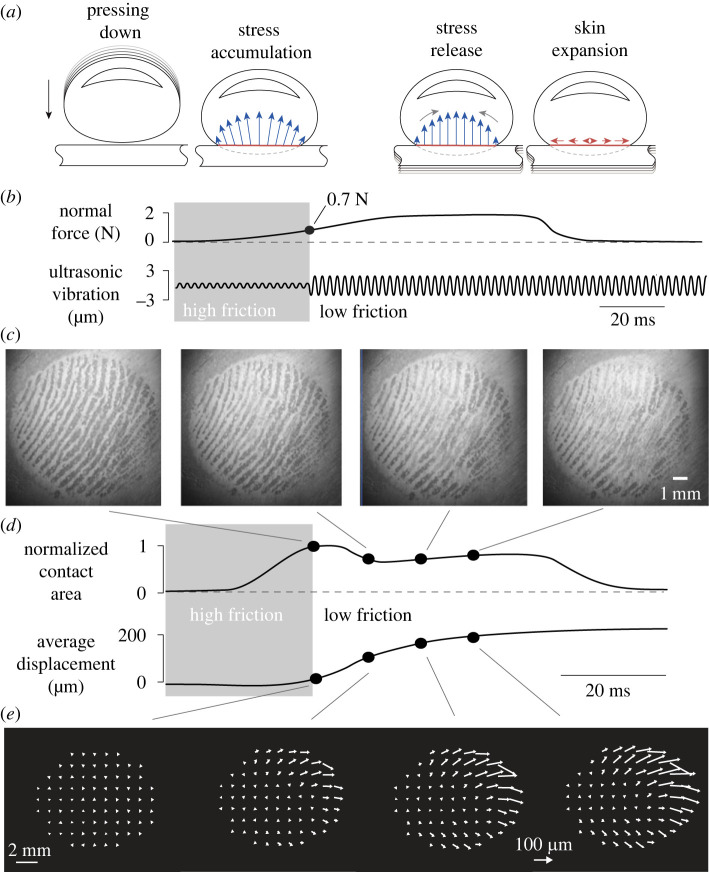
Typical trial. (*a*) Illustration of the dynamics during a normal exploration. While the finger contacts the plate, the skin expands outward, creating residual stress. This stress is released by rapid change of friction. (*b*) Evolution of the normal force and ultrasonic amplitude. The vibration amplitude changes when the normal force reaches 0.7 N. (*c*) Image of the real contact between the plate and the skin during the transition. (*d*) Temporal evolution of the contact area and the average skin movement. (*e*) Displacement fields show the expansion at a selected instant after the trigger.

In this paper, we show that the sensation of touching a virtual button arises when the ultrasonic waves release the elastic energy stored during pressing (see figure 1*a*,*b*). When the ultrasonic vibration is turned on, the skin slightly levitates (figure 1*c*) causing a decrease in real area of contact and a deformation of the skin (figure 1*d*). The deformation of the skin following the stress release spreads radially outward (see figure 1*e*).

## 2. Material and methods

### 2.1. Apparatus

**Figure 2.**
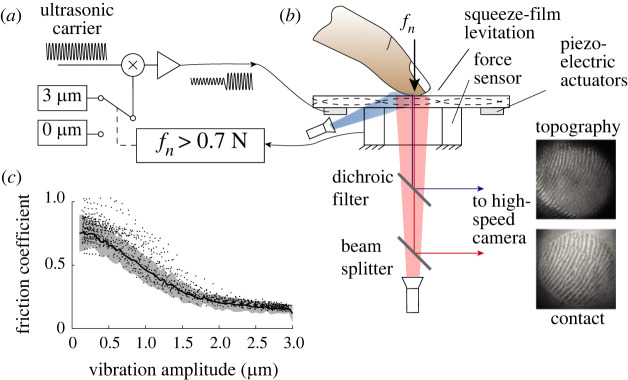
Experimental apparatus. (*a*) Signal processing. (*b*) Ultrasonic plate to modulate friction with high-speed imaging synchronously captures the contact image and the skin topography. (*c*) The friction coefficient is reduced with increasing vibration amplitude.

The experimental apparatus comprised two sub-modules: an optical path to image the contact and the skin topography, and a plate excited with ultrasonic bending waves to modulate the friction coefficient. The friction modulation section of the apparatus is illustrated in figure 2*a* and the optical arrangement to measure the contact is shown in figure 2*b*. The influence of the vibration on the friction of a sliding finger is shown figure 2*c*.

#### 2.1.1. Contact optical imaging

The custom optical system has two distinct illuminations of two different colours. The first illumination is a 450 nm blue light (Thorlabs M455L3) directed to the fingertip with a low incidence angle. The grazing light highlights the topography of the skin and provides robust information for tracking the features of the skin, including before the contact occurs. The second illumination is a 660 nm red light (Thorlabs M660L4) and is shone orthogonal to the surface of the glass. The light is emitted through a beam splitter which ensures coaxiality with the return path. This illumination uses frustrated reflection of the glass–air interface to highlight the micro-junctions where the asperities of the skin are in intimate contact with the plate [19]. In the absence of the finger, 4% of the incident light is reflected toward the sensor, but if part of the skin is in intimate contact with the glass plate, the reflection is frustrated, leading to a dark area on the image. A dichroic filter is used to spatially separate the two illumination sources so the two images are acquired by a single high-speed camera (Miro M110).

#### 2.1.2. Friction modulation device

To modulate friction in real time, we used an ultrasonic glass plate. The transverse vibrations levitate the skin away from the surface, reducing the number of micro-junctions and, therefore, reducing friction. The device used in the experiment measured 50 × 67 × 5 mm^3^ and vibrated in a 3 × 0 mode at a resonant frequency of 28.85 kHz. A piezoelectric sensor glued to the centre of the plate was used to measure the plate deformation in real time. The sensor, calibrated with an interferometer (IDS3010, Attocube, Munchen, Germany), gave a linear response within the entire ±4 μm amplitude range. The plate was mounted on an aluminium frame fixed on a 6-axis sensor (Nano43, ATI Industrial Automation, Norwalk, CT, USA). We optimized the force sensor placement to avoid large torques and cross-talk.

The piezoelectric actuator glued to the glass plate was driven at resonance by a ±200 V carrier signal, whose amplitude was modulated under computer control.

### 2.2. Participants

Twelve right-handed subjects aged from 22 to 35 years (3 females and 9 males) participated in the study. They were naive as to the purpose of the experiments and had no previous experience with haptic devices. None of them reported having any skin conditions or perceptual deficits. They gave their informed consent. The study was approved by the Comité de Protection des Personnes Sud Mediteranée ethics committee (2019-14-11-003). Finger images of four participants were acquired and processed to extract contact brightness and finger skin deformation.

### 2.3. Protocol

We used a randomized two-alternative forced choice (2-AFC) protocol where participants had to discriminate which one of the two stimuli moved similarly to a mechanical detent found in computer keyboards.

The reference stimulus had a medium friction level, maintained throughout the entire press and the comparison stimulus was a step change of friction whose average was the medium friction level. This medium friction level was obtained for a vibration amplitude of ±1.5 μm from base to peak. The six comparison signals were constructed by choosing between falling friction (where the amplitude was going from low to high) and rising friction followed by choosing one of the three levels of friction changes (low, medium, high) that correspond to an amplitude change Δ*A* of 1, 2 and 3 μm, respectively. As an example, for the falling friction condition, the three amplitude changes were constructed as follows: from 3 to 10^−3^ μm, from 2.5 to 0.5 μm and from 2 to 1 μm. The change of friction was triggered by measuring in real time the force amplitude, and if the force reached the threshold of 0.7 N, the controller would change the ultrasonic amplitude. The controller has a latency of 0.5 ms, which translates into a negligible jitter of the force threshold. During a pilot experiment, we measured the dynamic coefficients of friction when the vibration amplitude was modulated from 10^−3^ to 3 μm. When participants were steadily sliding across the plate, we found that the coefficient of friction varied from *μ* = 0.75 ± 0.14 down to *μ* = 0.12 ± 0.07.

Each stimulus was repeated 10 times for a total of 120 trials. The experiment lasted approximately 60 min. Participants could take a 5 min break every 15 min. To mitigate the learning effects, the participants were first shown the reference stimulus and a typical falling friction condition with a 3 μm amplitude variation. Participants wore headphones playing pink noise. A tone indicated the start of each trial and a double tone that they needed to answer.

To avoid any lateral movement during pressing, the finger was secured to a vertical linear guide. The palm rested on a support, which stabilized the participant’s hand, ensuring repeatable interaction.

### 2.4. Image analysis

The contact image and deformation image were recorded synchronously. We registered the images by computing the homogeneous transform from a known calibration object. The illumination drift was corrected using images without any finger. We recorded images at 1000 Hz 5 ms before and 40 ms after the friction change was triggered.

To capture the deformation of the skin, we tracked 800 features on the topography image with a key image at the instant of the trigger. These features were tracked inside the ellipse of contact using Lucas and Kanade optic flow algorithm. We computed a metric to extract the average displacement of the skin by taking the spatial average of the norm-2 of the displacement field *u*(*x*, *y*):

2.1 $$\bar{u}=\frac{1}{S}\int_{S}\|u(x,y){\|}_{2}\;dx\;dy,$$

where *S* is the contact area and *x*, *y* are the spatial coordinates.

### 2.5. Statistical analysis

The influences of friction change in direction or amplitude Δ*A* on contact area and skin displacement were assessed with analyses of variance (ANOVA). One-sample *t*-tests were used for *post hoc* comparisons when ANOVA showed a significant effect of one or multiple independent variables. The significance threshold used was 0.05.

## 3. Results

### 3.1. Perceptual experiment

**Figure 3.**
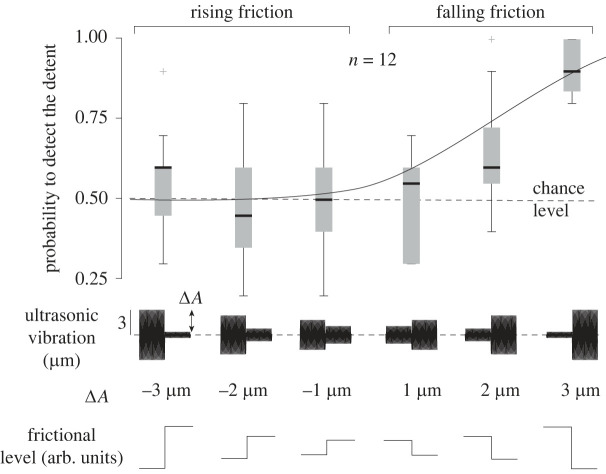
Probability of detecting the step change as a function of the direction of change (falling or rising) and of the amplitude of the step. Only sudden reductions of friction are perceived robustly. The error bars are over the probability of detection for the 12 participants.

The probability of detecting the comparison from the reference of the psychophysics experiment for all cases is shown in figure 3. The vibration amplitude and the direction of the frictional change had a significant effect on the perception of the keyclick (ANOVA, *F*_5,66_ = 13.4, *p* = 5 × 10^−9^ and *F*_1,70_ = 12.65, *p* = 0.0007, respectively). The responses follow a psychometric curve, with an unambiguous detection for rapidly decreasing friction with the highest amplitude variation of 3 μm. Participants responded at chance level for every rising friction condition, where the friction was changing from low to high, consistent with previous studies [9,10,20].

We fitted the results with a logit function and found that the 75% detection threshold was reached for Δ*A* = 2.5 μm. For reference, when actuating the plate at 2.5 μm, the sliding friction is reduced by ≈30%.

The psychophysical results clearly show that only a significant decrease in friction is perceptible, and the perception is unreliable when friction increases. We postulated in a previous paper that this specificity to the friction reduction was the effect of the sudden release of frictional stress at the interface [2]. The following section explores the contact mechanics associated with this perception of click.

### 3.2. Contact evolution

The contact images are used to extract a measure of the real contact area. This real contact area is the sum of all the junctions between the asperities of the skin and glass plate. The real area of contact is known to be linearly correlated to the frictional strength of the contact, therefore providing a measurement of the friction at the interface without resorting to force measurement during gross slippage [19,21]. A larger contact area has more junctions to adhere to the surface and therefore experiences more friction, assuming a constant shear strength of the interface.

For every trial, the increase in normal force causes an increase in the area of contact. The normal force typically reaches 2–3 N at the end of the press. We set the trigger when the force reached a 0.7 N threshold so that the friction modulation occurred at the early stage of the establishment of the contact. During reference trials, the friction was unchanged, and the contact brightness steadily increased by 39.7±54.1%. The increase of brightness can be explained by an increase of apparent contact area and normal force when the participant pressed their finger.

**Figure 4.**
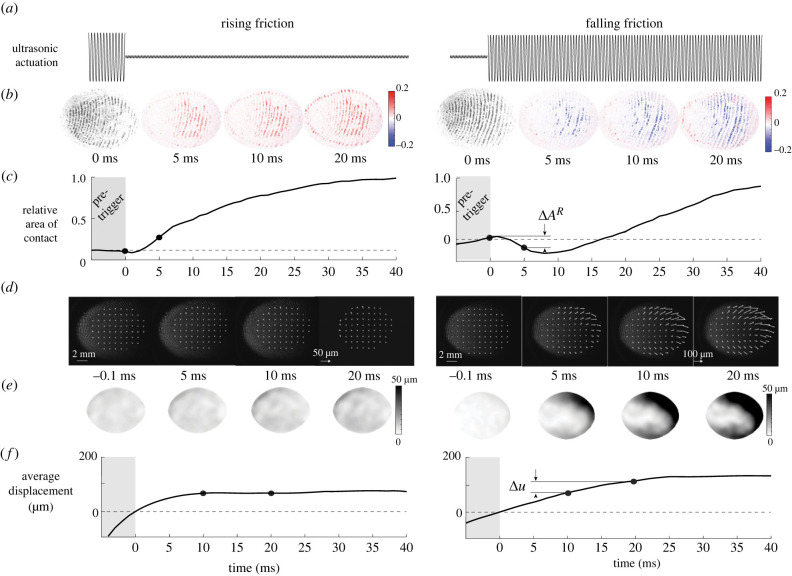
Typical data during two different trials, where friction is rising (left) and friction is falling (right). (*a*) Vibration amplitude negatively correlates with the value of the friction coefficient. (*b*) Contact images at selected instants of the deformation. The ultrasonic vibration decreases the number of asperities in intimate contact. (*c*) Evolution of the relative contact area. (*d*) Images and displacement field of the skin at the selected moment of the movement. (*e*) Norm of the displacement vector field. (*f*) Evolution of the median of the norm of the displacement field.

Significant differences in the evolution of the contact area during falling and rising friction conditions with respect to reference trials are found (ANOVA, *F*_2,477_ = 164.23, *p* < 0.001). During the transition from low to high friction, the area of contact has notable dynamics. Around 5 ms after the change of friction is triggered, the contact area rises sharply (figure 4*b*,*c* left), marking a lower levitation height and the formation of new junctions between the skin and the glass plate (M=41.60%, s.d.=56.22%, *t*_119_ = 8.11, *p* < 0.001).

During falling friction trials, where the friction changed from high to low, the contact area rapidly decreases within 5 ms after the transition (M=−13.54%, s.d.=20.87%, *t*_119_ = −7.41, *p* < 0.001) (see figure 4*b*,*c* right). This decrease demonstrates that the ultrasonic actuation reduces the number of junctions between the skin and the glass plate. The decrease of contact is positively correlated with friction reduction (Spearman'*s* coefficient = 0.123, *p* < 0.0001). Twenty milliseconds after this rapid reduction of contact, the contact rises again under the increase of normal force from the participant.

When inspecting individual trials, we also observed that the majority of falling friction trials shows that the losses of contact are concentrated in specific clusters that grow slowly, and the front of which propagates laterally. This effect might be due to the complex interaction between ultrasonic levitation and soft skin. Pockets of over-pressured air are nucleating and diffusing throughout the area of contact before finally reaching equilibrium. By contrast, during rising friction cases, contact increases over the entire finger surface.

### 3.3. Contact and skin displacement

In addition to the real contact area, we measured the displacement field of the skin. We observed the movement of patches of skin during the normal compression of the fingertip pulp. During reference trials, the ultrasonic vibrations are maintained at 1.5 μm causing an incomplete contact between the finger and the plate. The observed increase in contact area after 0.7 N is only due to the increase in finger pressure. The skin of the fingertip exhibits a lateral displacement during compression (*M* = 15.6 μm, s.d. = 17.1 μm), in line with previously reported findings [2].

**Figure 5.**
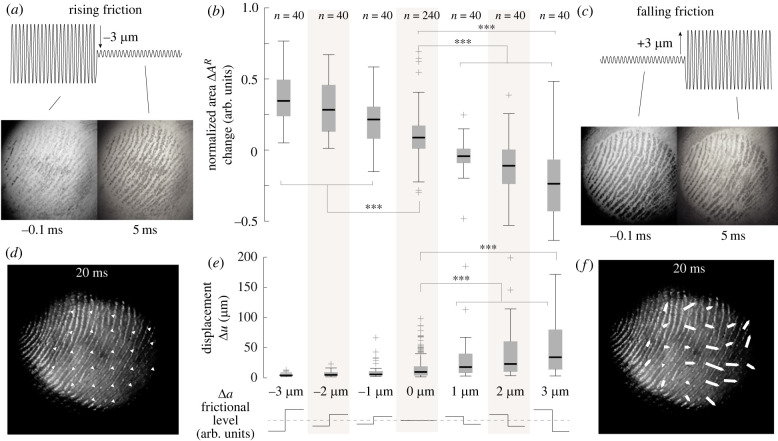
Effect of the amplitude changes on the contact and the deformation of the skin. (*a*) Contact area before and after a rise in friction. (*b*) The contact area changes negatively correlate with the amplitude variations. (*c*) Contact area before and after a rapid reduction of friction. The error bars represent the standard deviation across repetitions and subjects. (*d*) Deformation field after the amplitude change for rising friction. (*e*) The average deformation is close to zero when vibration increases and is positively correlated with vibration amplitude when the vibration changes are increasing. The error bars represent the standard deviation across repetitions and subjects. (*f*) Deformation field after the amplitude change for falling friction.

In the condition where friction increases, the movement of the skin only occurs before the trigger, since almost no friction is present at the initial moment of contact (figure 5*a*). After the rapid increase of friction, the growth of the contact area increases the friction and reduces the lateral displacement of the skin to almost zero in the 20 ms following the trigger (*M* = 7.4 μm, s.d. = 8.5 μm, *t*_119_ = 9.51, *p* < 0.001) (see figure 4*d*–*f* left and figure 5*d*). This sudden cease of lateral displacement can be explained by the high friction state that prevents any lateral movement of the skin.

In the falling friction condition, the contact initially develops with high friction. Consequently, only a slight lateral deformation of the skin is observed before the trigger. After the rapid reduction of friction, several detachment fronts nucleate in the area of contact, which causes a non-uniform movement of the skin (figure 5*c*). We measured a lateral displacement of the skin after the change of friction of 43.3 ± 54.1 μm (*t*_119_ = 8.77, *p* < 0.001) (see figure 4*d*–*f* right). The size of the change of lateral displacement followed a normal distribution, verified by a chi-square test rejecting the null hypothesis (*χ*^2^(1, *N* = 120) = 16.87, *p* < 0.001). The amplitude of the lateral movement significantly increases between friction conditions and for the reference stimulus (ANOVA, *F*_2,477_ = 49.63, *p* < 0.001).

The skin movement 20 ms after the trigger has an average displacement of 43.3 ± 54 μm. However, the displacement field is not uniform. The outer regions show a larger movement of the skin than the inner region, showing an expanding pattern similar to what is observed in [2] (see figure 5*f*).

Overall, the change of contact area decreases with increasing change of amplitude (ANOVA, *F*_6,553_ = 87.37, *p* < 0.001) (see figure 5*b*). Conversely, the global displacement shows a positive correlation with the amplitude of the change of ultrasonic vibrations (ANOVA, *F*_6,473_ = 21.39, *p* < 0.001) (see figure 5*e*).

### 3.4. Skin deformation causes perception

While the deformation of the skin is of the order of a few hundred micrometres, the relaxation of the skin after a reduction of friction is quick enough to generate a strain rate of roughly 5% per second compatible with the known detection threshold of rapidly adapting afferents [22].

**Figure 6.**
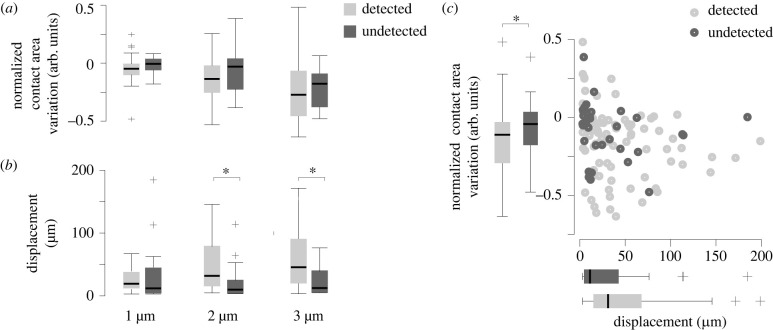
The relation between perception and skin mechanics for the four participants for which we processed images. (*a*) The variation of contact area has little effect on the detection (*n* = 40). (*b*) A stimulus perceived by the participants is likely to correspond to large skin deformation (*n* = 40). (*c*) Normalized contact area as a function of average displacement for every falling-friction condition (*n* = 40).

Using the data from the experiment, we can establish a causal connection between skin deformation and the perception of mechanical stimuli. The connection emerges when selecting only trials where the friction was decreased (falling friction). While the change of contact area pre- and post-trigger was not significantly different when the participant detected the stimuli or not (figure 6*a*), the amount of deformation of the skin had an unambiguous explanatory power over the probability of detection by the participant (figure 6*b*, Spearman'*s* coefficient = 0.123, *p* < 0.01).

The trials where the stimulus was not detected, but the amplitude was 3 μm, correspond to near-zero changes in the contact area and negligible deformation (see figure 6*c*). Conversely, the trials where the stimuli were correctly detected are associated with larger contact area changes and skin deformation.

### 3.5. Model predictions

**Figure 7.**
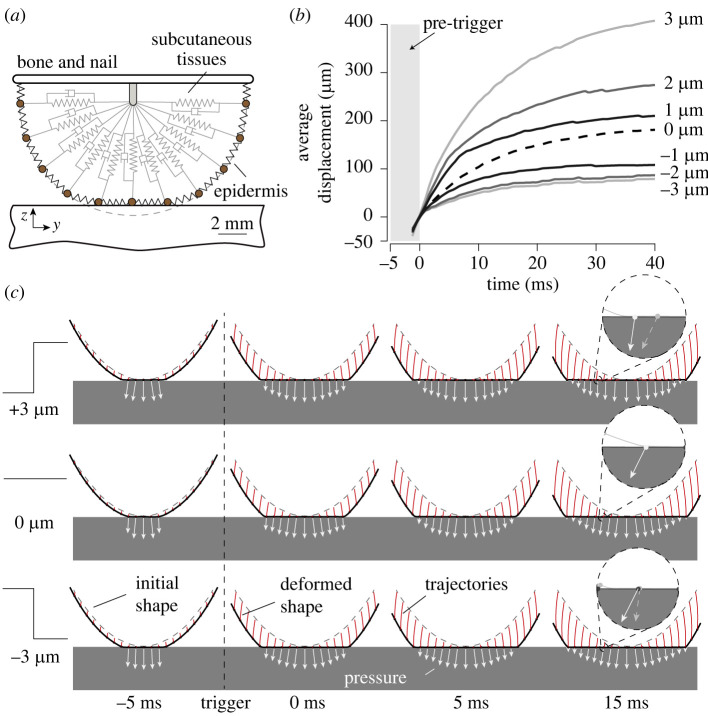
(*a*) Finite-difference model of the fingertip pulp under contact with finite friction. (*b*) Simulated lateral average displacement of skin patches for reference friction (dashed line, 0 μm), falling friction (1, 2, 3 μm) and rising friction (−1, −2, −3 μm). (*c*) Simulated finger profile with a 3, 0 and −3 μm friction change. The white arrows represent the accumulated stresses on the surface, and the red lines correspond to the features’ trajectories.

To better understand how the skin deforms after a sudden change of friction, we modelled the biomechanics of the fingertip under frictional contact. We used a finite-difference time-domain scheme to capture the deformation of the skin, viscoelasticity and local friction behaviour at the interface. The model is identical to the one used in [2]. We simulated the interaction between the finger skin and the plate surface under seven friction changes from −3 to 3 μm with a normal force of 4 N applied to the bone element (see figure 7*a*).

The rising friction case has a similar mechanical behaviour to the reference, which sees no change in friction. Because of the low friction, the elements start to expand laterally before the mechanical detent, when the friction is small. Then when we increase friction, the lateral component of the interfacial pressure increases and locks the elements in place. The simulated median displacements are shown in figure 7*b*. The curves flatten in all conditions, but despite a saturation, the median of the average displacement metric is eight times higher in the maximal falling friction condition compared to the rising friction condition.

Simulation results are shown in figure 7*c* where individual displacements of skin patches appear in red, and the stresses at the interface appear in white. In the falling friction condition, the lateral component of the interfacial pressure decreases significantly after the friction change, which happens at 0 ms. This release of mechanical stress results in significant lateral deformation of the skin.

## 4. Discussion

The skin of our fingertip is highly deformable and, therefore, can conform to a wide range of shapes and surfaces [23,24]. During the first instant of contact, friction plays a critical role in the way the skin will deform [1,2]. If friction is low, the skin will expand laterally and a negligible amount of tangential stress will be stored. Conversely, when friction is high, the skin grips to the surface when it makes contact, blocking its tangential expansion and storing elastic energy.

Surprisingly, this work shows that a sudden release of elastic energy by rapidly reducing friction can be perceived. Effectively this rapid change of friction is analogous to changing the boundary condition at the contact from fixed to a roller. A similar change of boundary condition arises when operating real buttons [13], which might explain why we perceive these changes of friction as a button click. The change in friction produces a notable lateral expansion of the skin, which takes less than 20 ms to fully develop. The time response is consistent with known values of the relaxation of the skin [25] suggesting that this duration is limited by the viscoelastic behaviour. The brief deformation of the skin can be sufficiently large to produce conscious sensations.

The ultrasonic vibration excites the skin in a range of frequencies and deformations that do not stimulate the mechanoreceptors in the skin. Yet participants reported perceiving the event, suggesting that the mechanical deformation of the skin, due to the release of friction, is sufficient to trigger a sensation. Participants reported that they felt they pressed a mechanical button, although no significant normal movement of the plate was recorded. Our hypothesis behind this association is that the detent that we feel when interacting with a real button produces a similar release of elastic energy to what is found when interacting with a mechanical button. Because the release of energy is congruent to the user’s action, this sensation of mechanical detent after a reduction of friction is perceived as the activation of a mechanical button.

The salience of the illusory button directly connects to the size of the friction change. The larger the friction change, the larger the skin deformation and the easier it is to detect the stimulus. Since the stored energy during compression is limited by the mechanics of the skin, there is an upper bound on the strength of the stimuli. In the present study, we focused our attention on purely normal exploratory motions, where the participant could only press on the plate since their finger was guided by a vertical linear guide. Therefore, we recorded only an insignificant amount of lateral force. However, we could increase the strength of the stimuli by storing additional elastic energy in the gross lateral deformation of the fingertip during compression, using surface-haptic devices that provide active forcing, such as in [26,27]. A lateral deformation at the initial instant of skin deformation could lead to potent sensations and salient click.

The observed skin deformation is of the order of a few tens of micrometres and yet it leads to a robust perception. We postulate that such a small change is perceivable because the deformation is quick enough to stimulate the rapidly adapting afferents. We showed that the expansion of the skin is a fundamental factor of our estimate of friction during the moments following the initial contact [2]. However, the discrimination of friction on initial contact is challenging, and a proper estimate of friction is made when there is a lateral motion of the skin on the surface [28].

## 5. Conclusion

In this study, we showed that micrometre-scale ultrasonic vibrations, which are not perceivable by touch, can create a distinct tactile stimulus resembling a button click when the friction suddenly decreases. The stimulus is created by an indirect stimulation of the skin: a near-field levitation effect reduces the friction and allows the skin to expand. To observe this skin expansion, we developed a custom optical apparatus that can simultaneously monitor the change in the real contact area—connected to the frictional strength of the contact—and the deformation of the skin. The observed skin expansion when pressing on the surface is at the root of the perception since larger deformation leads to an improved probability of detection by participants.

The perception of friction is central to the appreciation of materials and the motor control of grasp. Taken together, the observations presented in this paper allow us to postulate that the tactile perception of friction when actively interacting with our environment is initially caused by the lateral expansion of the skin.

## Data Availability

Data from this study are available at the open-access 4TU.ResearchData Repository (https://doi.org/10.4121/21220352.v1). Source code of the finger skin model data has been deposited in GitLab (https://gitlab.tudelft.nl/lwillemet/finger-model).
